# Mr. George's Panel Statistics

**Published:** 1914-02-14

**Authors:** Thomas Johnston

**Affiliations:** Hon. Sec. The Sussex Non-Panel Union, Federated with the "National Medical Union," 91 Carlisle Road, Hove


### National Medical Union.

*To the Editor of* The Hospital.

### Mr. George's Panel Statistics.

*To the Editor of* The Hospital.

Sir,—Mr. Lloyd George on the 6th inst. stated there were over 20,000 doctors on the panel, including those on more than one panel. Brighton is one Insurance area, and has seventy-six on the panel. Hove is another Insurance area, and has seventy-one on the panel. For Mr. Lloyd George's statistics this makes 147 doctors on the panel. The towns adjoin, and there are only eighty-seven doctors on the panel for Hove and Brighton, sixty of whom are on both panels.

This kind of duplication occurs all over the United Kingdom. It is unfair and misleading to dilate statistics by counting duplicates, and sixty out of 147 is a very large percentage. If he said 12,000 instead of 20,000 he would be much nearer the truth. I remain, yours faithfully,

Thomas Johnston, Hon. Sec.

The Sussex Non-Panel Union, Federated with the "National Medical Union," 91 Carlisle Road, Hove, February 10, 1914.

